# Molecular Markers for Biomass Traits: Association, Interaction and Genetic Divergence in Silkworm *Bombyx mori*

**Published:** 2007-05-30

**Authors:** Appukuttannair R Pradeep, Anuradha H Jingade, Raje S Urs

**Affiliations:** Seribiotech Research Laboratory, Central Silk Board, CSB Campus, Kodathi, Carmelram. P.O; Bangalore, Karnataka, India. Pin - 560 035

**Keywords:** Biomass traits, ISSR marker-trait association, interaction, plasticity, genetic distance, Bombyx mori

## Abstract

Improvement of high yielding, disease resistant silkworm strains became imminent to increase production of silk, which is a major revenue earner for sericulturists. Since environment interacts with phenotype, conventional breeding did not result in commendable yield improvement in synthetic strains of silkworm, *Bombyx mori*. Identification of DNA markers associated with different economically important biomass traits and its introgression could assist molecular breeding and expression of stabilized high yielding characters, but genetic basis of most quantitative traits in silkworm is poorly understood due to its polygenic control. Correlation analysis (R = 0.9) revealed significant interrelation among biomass traits *viz*., larval duration (TLD), larval weight (LWT), cocoon weight (CWT), shell weight (SWT), shell ratio (SR) and floss content. PCR using inter simple sequence repeat (ISSR) primers revealed 92% polymorphism among 14 tropical and temperate strains of *B. mori*, with average diversity index of 0.747. Stepwise multiple regression analysis (MRA) selected 35 ISSR markers positively or negatively correlated with different biomass traits, illustrated polygenic control. ISSR marker 830.8_1050bp_ was significantly associated with LWT, CWT, SWT, SR and floss content, indicated its pleiotropic role. Two ISSR markers, 835.5_1950bp_ and 825.9_710bp_ showed significant association with floss content and TLD. These markers were segregated in F_2_ generation and Chi-square test confirmed (χ^2^ = ~45; *P* < 0.05) its genetic contribution to the associated biomass traits. Strains, with both positively and negatively correlated markers, had intermediate mean value for biomass traits (eg. SWT = 0.17 ± 0.014 g in *GNM* and *Moria*) indicated interaction of loci in natural populations. Low yielding Indian strains grouped together by Hierarchical clustering. Chinese and Japanese strains were distributed in the periphery of ALSCAL matrix indicated convergence of genetic characters in Indian strains. Average genetic distance between Chinese strains and Indian strains (0.193) significantly (*P* < 0.01) varied from that between Chinese and Japanese strains. Interaction of loci and allelic substitutions induced phenotypic plasticity in temperate *B. mori* populations on tropic adaptation in India. These outcomes show possibility to combine favorable alleles at different QTL to increase larval, cocoon and shell weight.

## Introduction

Sericulture or cultivation of silkworms is an agro industry, producing commercially valuable silk on which scores of farmers of tropical and temperate Asian countries rely for their revenue. Domesticated silkworm *Bombyx mori* (Insecta: Lepidoptera: Bombycidae) is monophagous and feeds exclusively on leaf of mulberry, a hardy plant belongs to the genus *Morus* (Family: Moraceae). Silkworm germplasm encompass around 3000 genotypes having its origin in temperate and tropical countries (Nagaraju et al. 2000). *B. mori* gene pool is broadly categorized in to low yielding and high yielding strains. Low yielding strains are adapted to tropical conditions and are non- diapausing while high yielding strains adapted to temperate climate and undergo embryonic diapause. High yielding strains have higher cocoon weight, cocoon-shell weight, shell ratio and better yarn qualities in comparison to low yielding strains (www.silkgermplasm.com) but are highly susceptible to diseases. India, being a tropical country utilizes low yielding native strains, and breeds developed from Japanese and Chinese strains of *B. mori* for silk production. Silkworm breeding strategy is aimed at developing vigorous breeds and hybrids to meet twin demand of high survival and high production of quality silk. Though phenotypic characters depict variation, interaction of environment modifies its expression. Therefore, gene tagged breeding would be a promising approach to combine best quality of temperate high yielding strains with tropical disease resistant ones. Since most quantitative economic characters are controlled by interactions between multiple genes and environment, identification of gene(s) associated with a specific character is an enigma. Genetic analysis of quantitative traits became possible due to availability of large number of molecular markers to which QTL is associated. Genetic characterization of various silkworm strains of diversified phenotype and identification of gene markers for each of the economically important characters could contribute to develop strategy for future breeding programs using marker-assisted selection. Genes that contribute to naturally occurring variations in quantitative traits of *B. mori* strains may not vary in the mapping population too. Hence, association of markers with different traits and its distribution in natural populations are to be detected. Molecular marker systems like Random Amplified Polymorphic DNA (RAPD; Nagaraja and Nagaraju, 1995; Chatterjee and Pradeep, 2003), Restriction Fragment Length Polymorphism (RFLP; Sethuraman et al. 2002), microsatellites (simple sequence repeats; SSR; Prasad et al. 2005) and inter SSR (ISSR; Reddy et al. 1999; Pradeep et al. 2005) highlighted the utility of molecular markers in silkworm fingerprinting and analysis of marker-trait association. These potential marker systems and single nucleotide polymorphism (SNP) markers were also used to generate molecular maps of *B. mori* (Goldsmith et al. 2005; Nagaraja et al. 2005; Yamamoto et al. 2006).

ISSR products resolved on agarose gel are dominant markers and the system offers rapid production of a large number of markers in cost-effective manner. ISSRs are DNA fragments located between adjacent, oppositely oriented microsatellites amplified by PCR using microsatellite core sequences and a few selective nucleotides as a single primer. As short repeats are ubiquitously distributed in eukaryotic genome, single primers of di-, tri-, tetra- and penta nucleotide simple sequence repeats are employed for amplification of markers. ISSR markers, evolve faster as they are genomic regions with microsatellites that exhibit variable mutation rates and high level of polymorphism (Schlotterer, 2000), due to DNA polymerase slippage or DNA double strand breakage (Strand et al. 1993; Jankowski et al. 2000). After initial identification of ISSRs in humans (Zietkiewicz et al. 1994), its greater usefulness in fingerprinting has been established in different organisms including plants (Nagaoka and Ogibara, 1997; Agaki et al. 1996; Deshpande et al. 2001) and insects (Ehtesham et al. 1995; Reddy et al. 1999; Abbot, 2001; Chatterjee et al. 2004; Vijayan et al. 2006; Pradeep et al. unpublished). ISSR markers are usually located in non-coding regions and are selectively neutral. Because ISSR primers generate multi locus fingerprinting profile, ISSR analysis has been applied in studies involving genetic identity, parentage, clone and strain identification as well as gene mapping studies (Vogel and Scolnik, 1997). Considering these advantages of ISSR primers, this marker system was used to identify molecular markers associated (not linked) with biomass traits and to analyze genetic variability among few strains of *B. mori*.

Association of molecular markers with different economic traits or disease resistance was studied mostly in crops such as chick pea (Ratnaparkhe et al. 1998), rice (Hussain et al. 2000) and maize (Domeniuk et al. 2002). Molecular markers for antibody response in chickens (Yonash et al. 2000) and gene for larval growth in *Drosophila* (Becker et al. 2001) were also reported. Due to the economic importance of silkworms and need for high yielding disease resistant strains, conventional breeding techniques has to be supported by directional selection utilizing yield associated molecular markers. Hence investigations on association of various molecular markers with different yield attributes had initiated in silkworms (Sethuraman et al. 2002; Chatterjee and Mohandas, 2003; Chatterjee and Pradeep, 2003; Pradeep et al. 2005; Gaviria et al. 2006) (Table 1). Since each quantitative trait is under regulation of different genes, their associations with different traits have yet to be established. Association of molecular markers with different traits was studied in different organisms using methods such as MRA, bulk-segregant analysis (BSA) and discriminant function analysis (DFA). While MRA provided statistical association of markers, based on its correlation with traits (Virk et al. 1996; Yonash et al. 2000), BSA could identify markers for a specific trait from segregating population (Michaelmore et al. 1991). On the other hand, DFA used a group co-variance matrix, adopting stepwise selection of independent variables. DFA facilitated identification of a molecular marker that revealed geographical isolation of Japanese strains of *B. mori* from mainland (Sino-Russian-Indian) populations (Chatterjee and Pradeep, 2003). In the present investigation, we identified polymorphic ISSR markers related with different quantitative biomass traits. Association of these ISSR markers with different biomass traits was established through single factor ANOVA and its genetic contribution was confirmed by Chi-square test in F_2_ generation. Effect of interaction between markers on phenotype variability in natural population was established by significance test. Genetic divergence among different strains within a gene pool of *B. mori* of India, China and Japan was documented by Hierarchical cluster analysis using three statistical measures.

## Materials and Methods

### Genetic material and DNA extraction

Fourteen strains of *B. mori*, originating from India, China and Japan used in this study, were maintained at Central Sericultural Germplasm Resource Centre, Hosur, Tamil Nadu, India where strains were reared for more than 10 years at standard rearing conditions of 25 ± 2 °C temperature and 75 ± 3% relative humidity. Phenotypic data collected from three replications of the rearing (*n* = 30 each) is given in Table 2. To study inheritance of markers, two crosses were made between low yielding strains *Pure Mysore/C’nichi* females and high yielding strain *NB1* (male). F_1_ generations were raised and moths were allowed for sister-brother crossing (self mating) to develop F_2_ generation of both the crosses. Phenotypic data of each F_2_ individual was collected (*n* = 32). Genomic DNA of different *B. mori* strains (*n* = 30 individuals each) and F_2_ generation individuals was extracted from moths by phenol:chloroform: isoamyl alcohol method (Suzuki et al. 1972). DNA was dissolved in TE (Tris-EDTA) buffer (pH 8.0) and diluted and quantified to a concentration of 10 ng per micro Liter against standard uncut lambda DNA (10 ng/micro Liter).

### PCR conditions and amplification

One hundred ISSR primers (Set No.9: procured from University of British Columbia (UBC), Vancouver, Canada) were screened initially and twenty five of them produced robust reproducible bands with genomic DNA of 14 strains of *B. mori* (Table 2). PCR amplification was performed in 20 microLiter reaction mixture of 10 mM tris-HCl buffer, 2.0 mM MgCl_2_, 0.2 mM each dNTPs and 0.12 units of *Taq* DNA Polymerase (Fermentas Life Sciences, Vilnius, Lithuania) with 40 nanogram of template DNA and 0.15 micromole ISSR primer. All reactions were performed in a DNA Engine (Peltier Thermal Cycler PTC 200; MJ Research Inc., Mass., U.S.A). PCR conditions followed were initial denaturation at 94 °C for 2 minutes, followed by 35 cycles of 94 °C for 30 seconds, 50 °C for 30 seconds and 72 °C for 2 minutes. Final extension was at 72 °C for 10 minutes. PCR products along with a standard molecular weight marker (Mass Ruler, Fermentas Life Sciences, Lithuania) were resolved on 1.5% agarose gel in 1× TBE (Tris-Boric acid-EDTA) buffer. Gels were stained with ethidium bromide (0.5 microgram/mL) and UV illuminated gels were photographed using a gel documentation system (Syngene Corporation, UK). Reproducibility of robust bands was confirmed by two subsequent reactions.

## Statistical Analyses

Data generated by ISSR primers were used for analysis using the program SPSS v 11.5 (M. J. Norusis, SPSS Inc., Chicago). Banding pattern generated by each primer was scored into a matrix with presence of amplification product as “1” and absence as “0” and this binary matrix was used for analysis.

Biomass traits considered for this study were total larval duration from hatching to initiation of spinning (TLD), maximum weight attained by final instar larva (LWT), cocoon weight (CWT), cocoon shell weight (SWT), shell ratio (SR % = SWT/CWT × 100), outer loose layer of silk over the cocoon or floss (%) and reeling silk waste (%). Differences between mean estimates of traits among 14 strains were assessed by ANOVA. Interrelation between different traits was assessed by correlation analysis. Multiple regression analysis (MRA) was used for identification of markers associated with different biomass traits with molecular markers as independent variable and biomass trait estimates as dependent variables. Stepwise variable entry and removal used in MRA examined the variables at each step for entry or removal. MRA used the model for regression equation with *F* values of 0.045 and 0.099 as limiting frame for stepwise selection and rejection of the independent variable (Affifi and Clark, 1984). *Beta* statistics was calculated for each marker and is defined as standardized regression coefficient = *BSx/Sy*, where *B* is regression coefficient, *Sx* and *Sy* are the standard deviations of independent (*x*) and dependent (*y*) variables (Affifi and Clark, 1984). Student’s *t*- test was performed to test significance between mean trait estimates of strains where specific markers were present and absent.

Single factor ANOVA (SFA) was performed to establish association of markers with biomass traits of different strains as well as of F_2_ generation individuals. The procedure produces a one-way analysis of variance for a quantitative dependent variable (trait) by a single factor (independent) variable (molecular marker). Single marker analysis (SMA) was performed with MRA selected markers as the classifying variable to identify QTLs associated with biomass traits in F_2_ generation. Chi-square (χ^2^) test was performed to examine goodness-of-fit between marker-locus contributions in F_2_ generation. Effect of interaction of MRA selected markers on its association with different traits was assessed by analyzing level of significance (Students’ *t*- test) in difference between estimates of each trait.

In order to analyze genetic divergence data developed from dominant ISSR markers, genetic similarity coefficients among 14 strains were estimated from the binary data by Heirarchical cluster analysis using Jaccard measure, Dice measure and Sokal and Sneath measure. Jaccards’ coefficient was GD_J_ = 1−[*N*_11_/(*N*_11_*+N*_10_*+N*_01_)], Dice coefficient was GD_D_ =1−[*2N*_11_/(*2N*_11_*+N*_10_*+N*_01_)] where *N*_11_ is the number of bands present in both individuals, *N*_00_ is number of bands absent in both the individuals, *N*_10_ and *N*_01_ are number of bands present only in the individual and *N* represents the total number of bands. Sneath and Sokal, (1973) coefficient for genetic distance between genotypes i and j (Dij) was determined by Dij = 1−Sij = 1−[a + d/(a + b + c + d)] where Sij = similarity coefficient; a = number of matches 1,1; b = number of matches 1,0; c = number of matches 0,1 and d = number of matches 0,0. Genetic distance was calculated as (1-Similarity coefficient). Dendrograms were resolved from similarity matrices to compare genetic distance among strains based on different algorithms. In order to analyze distribution of silkworm genotypes from India, China and Japan, multidimensional scaling of ISSR data from 14 strains was done using ALSCAL program. In this method, a dissimilarity matrix was created using Euclidean distance and was used for stimulus configurations of the data using the classical Young-Householder multidimensional scaling procedure (Young et al. 1984; Young and Harris, 1990).

## Results

Mean estimates of biomass traits, country of origin and diapause behavior of 14 different strains of *B. mori* is given in Table 2. Among the strains, significant (ANOVA; *P* < 0.005) variation was observed within estimates of biomass traits such as LWT, CWT, SWT, SR, floss as well as silk waste.

### Interrelation between biomass traits

Correlation analysis showed positive correlation (R = 0.916) among mean estimates of LWT, CWT, SWT and SR (Table 3). SWT (R = 0.923) and SR (R = 0.742) showed significant increase with increase in CWT. Increase in SWT (R = 0.554) and SR (R = 0.607) showed highly significant (*P* < 0.001) increase with TLD but this relation was not apparent with other parameters. Floss content showed negative relation with increase in LWT and CWT (R = −0.786). Quantity of silk waste did not show significant relation with larval characters but showed negative correlation (average R = −0.593) with cocoon characters.

### ISSR polymorphism among *B. mori* strains and molecular markers for biomass traits

Twenty five ISSR primers were used for amplification of genomic DNA of 14 strains of *B. mori*. A total of 252 bands were generated, of which 92% (range 66.67–100%) were polymorphic (Table 4). Size of amplification products ranged from 500 bp to 3500 bp. Dinuleotide repeats, (AG)_8_C, (GA)_8_A, (AC)_8_C, (TG)_8_G, (AG)_8_YC, (CT)_8_RC, trinucleotide repeat, (AGC)_6_ and pentanucleotide repeat, (GGGTG)_3_ produced 100% polymorphism. Average diversity index (DI) was 0.747 for the ISSR primers used, of which UBC841 ((GA)_8_YC) showed highest (0.943) and UBC 862 ((AGC)_6_) showed lowest diversity index value (0.103). One of the low yielding strains, *C’nichi* and five high yielding strains showed presence of exclusive PCR products (Table 4).

Based on binary matrix of ISSR profile, step wise MRA identified 35 ISSR markers associated with different biomass traits. Details of MRA and *beta* statistics with significance are given in Table.5. In the first step, MRA selected ISSR marker 830.8_1050bp_ for LWT, CWT, SWT and SR. On linear regression, this marker was negatively correlated with increase in estimates of these parameters (R^2^ = ~0.8). Subsequently, MRA selected 851.1_1700bp_ for LWT (R^2^ = 0.930), 810.2_1350bp_ for CWT (R^2^ = 0.948) and 834.11_900bp_ for shell weight (R^2^ = 0.925) and shell ratio (R^2^ = 0.826). For TLD, 825.9_700bp_ was selected initially (R^2^ = 0.738), followed by 835.11_1000bp_ (R^2^ = 0.738). The marker, 825.9_700bp_ was present in low yielding strains except in *Pure Mysore* and absent in most of the high yielding strains. Highest number of markers (seven) was selected for floss content, of which four were selected with negative correlation and three with positive correlation. The marker, 830.8_1050bp_ was selected in the first step for floss content also but found as positively correlated (R^2^ = 0.631). For silk waste, 881.4_2000bp_ was selected in the first step and it showed weak negative correlation (R^2^ = 0.557). All together, 16 markers were positively correlated with increase in estimates and 19 markers were negatively correlated (Table.5).

## Association of Markers with Traits

**Figure 1:**
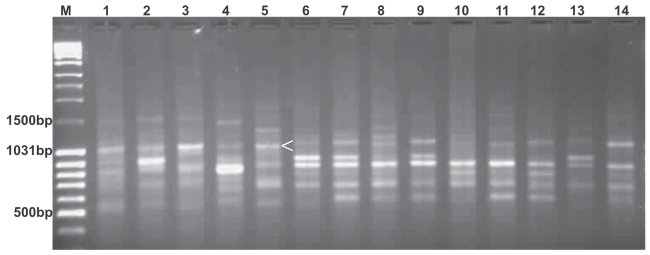
ISSR profile generated from genomic DNA of 14 strains of *B. mori* using the primer UBC 830. Arrow shows the presence of marker (830.8_1050bp_) in low yielding strains. 1–14 represents strains as listed in Table 2. M- molecular marker (Massruler, Fermentas).

Student’s *t*- test confirmed significance (*P* < 0.005) in variation between phenotype estimates of strains showing presence and absence of marker associated with each trait (Table. 6). The marker 830.8_1050bp_ showed highly significant (*P* < 0.0003) association with low estimates of LWT, CWT, SWT, SR and with high estimate of floss content (*P* < 0.005). This marker was present in low yielding strains of *B. mori* (*Nistari, C’nichi, Pure Mysore*, *GNM* and *Moria*) and was conspicuously absent in high yielding strains (Fig. 1). Of the other markers, 835.9_1050bp_, 836.4_2300bp_ and 835.5_1950bp_ showed significant (*P* < 0.02 to 0.002) positive association with high estimates of TLD, LWT and floss content respectively. All other markers showed significant negative relation with different traits (Table 6). In F_2_, MRA selected markers segregated in 1:1 ratio, except 835.5_1950bp_, in 3:1 ratio. Single factor ANOVA showed highly significant (*P* < 0.000) association of 830.8_1050bp_ with LWT, TLD, CWT, SWT, SR and floss content of different strains and in F_2_ population (Tables 7A & B). Significantly high Chi-square values were observed in case of 830.8_1050bp_ and 836.4_2300bp_ (Table 8). While 830.8_1050bp_ showed skewed ness towards low yielding female parent *Pure Mysore* with regard to different biomass traits, 836.4_2300bp_ skewed towards high yielding male parent *NB1* with regard to LWT.

Effect of interaction of two markers associated with a specific character was analyzed by comparing estimates of traits of different strains using *t*-test. Mean values of TLD, CWT, SWT, silk waste and floss content of strains either with a negatively correlated marker, or with a positively correlated marker and strains with both the negative and positive markers are given in Table 9. *B. mori* strain *GNM* with both 830.8_1050bp_ and 810.2_1350bp_ had intermediate CWT (1.19g), which showed significant (*P* < 0.007) variation from CWT of strains with 830.8_1050bp_ alone but did not differ significantly from those with 810.2_1350bp_. In shell weight and silk waste, intermediate values showed significant (*P* < 0.005) variation from high and low estimates whereas in floss content, intermediate value of *C’nichi* significantly (*P* < 0.06) varied from low floss content strains but did not vary from high floss content strains (*P* < 0.164). LWT of strains having marker 830.8_1050bp_ was 2.265 ± 0.378g whereas that of strains with 836.4_2300bp_ was 3.987 ± 0.153g. No strains used had both the markers (830.8_1050bp_ and 836.4_2300bp_) together. F_2_ individuals (of *Pure Mysore* × *NB1* cross) in which both these markers were present had LWT of 2.914 ± 0.424 g (equivalent to mid-parent value), which was significantly (*P* < 0.0001; Student’s *t*-test) higher than LWT (2.314 ± 0.359 g) of individuals without these markers. TLD of the strain *HU204,* which had both 825.9_710bp_ and 835.11_1050bp_, was higher (631 hours) than other groups.

## Genetic Divergence Between Low Yielding and High Yielding Strains

**Figure 2:**
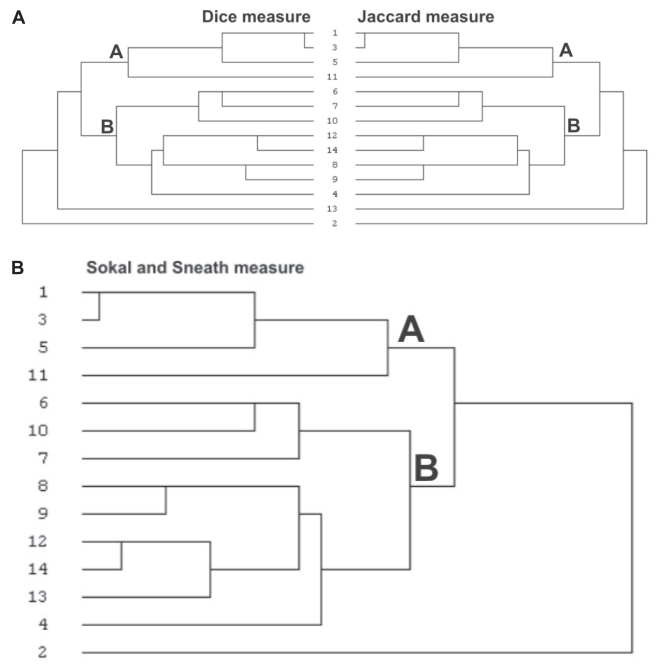
Dendrograms realized from the similarity coefficient calculated using (2.A) Jaccard measure, Dice measure and (2.B) Sokal and Sneath measure based on ISSR profile generated from genomic DNA of 14 strains of *B. mori*.

**Figure 3:**
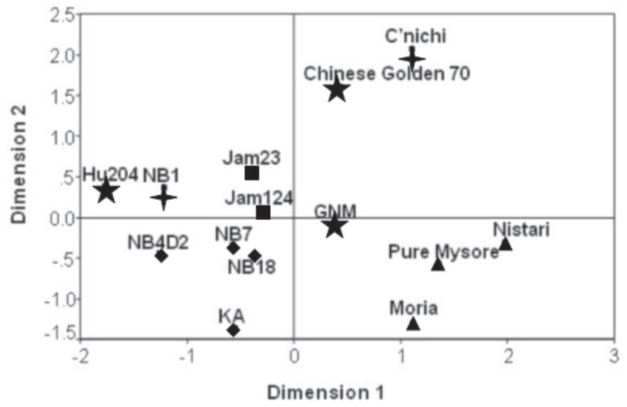
Distribution of 14 strains of *B. mori* on a two dimensional plot generated from Euclidean distances based on ISSR profile using ALSCAL multidimensional scaling. ▲Original Indian strains; ◆evolved from Japanese parents; ★Chinese strains; 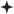 Japanese strains; ■Indian strains but parentage not known.

Polymorphic profile generated by ISSR primers from 14 different strains of *B. mori* (Table 4) was further analyzed by Hierarchical clustering. Three different algorithms *viz*., Jaccard measure, Dice measure and Sokal and Sneath measure were used to evaluate genetic relations among the 14 strains. Jaccard and Dice measures clustered strains in similar pattern but were different from grouping by Sokal and Sneath measure (Figs. 2A and B). Three Indian low yielding strains *Nistari*, *Pure Mysore* and *Moria* were grouped (A) together by all three measures. Group B comprised high yielding exotic strains of *B. mori*. All the measures isolated *C’nichi* from other strains. Dissimilarity matrix showed *Nistari* and *Pure Mysore* (0.108) as genetically closer strains and *C’nichi* as genetically distanced (0.667) strain from others (Table.10). Average genetic distance between Chinese and Indian strains (0.193) calculated using Sokal and Sneath measure significantly (*P* < 0.01) varied from that between Chinese and Japanese strains. Genetic distance between Chinese and Japanese as well as Indian and Japanese strains did not vary significantly. Multidimensional scaling of all 14 strains based on Euclidean distance showed grouping of three pure Indian strains, *Nistari*, *Pure Mysore* and *Moria* together and clustering of evolved strains separately. Most of the Japanese and Chinese strains were distributed in periphery of matrix (Fig. 3).

## Discussion

Biomass traits showed significant variability among different strains of silkworm, *B. mori*. Correlation matrix showed high coefficient value (>0.9) between larval weight and cocoon/shell weight indicated contribution of larval weight to formation of cocoon (pupa and its shell). Total larval duration contributed significantly to increase in cocoon and shell weight. In silkworms, larva is the only feeding stage in the life cycle and it accumulates energy for all life stages and contributes to formation of cocoon, pupa and moth as well as reproductive processes. In insects, critical larval weight together with larval duration accomplishes endocrine—mediated metamorphic processes (Pradeep et al. 2000; Truman 2005) but the process is under genetic control (Dubrovskaya et al. 2004). Correlation between quantitative traits and biochemical parameters had reported earlier in *B. mori* (Shibukawa et al. 1986; Chatterjee et al. 1993). Significant correlation among biomass traits reflects interrelation among physiologically important processes. Hormones coordinate multiple developmental and physiological processes and are major determinants underlying phenotypic integration (Flatt et al. 2005). High shell weight is accompanied by low floss content indicated that silk formed by the larvae is utilized to its maximum for shell formation in high yielding strains. Silk waste is determined after reeling of cocoons, which include mechanical processing that causes more wastage of filament. This may be the reason for lack of correlation of silk waste with biomass traits.

ISSR primers showed large diversity index (DI) of 0.747, of which dinuleotide repeats revealed higher level of diversity among the strains. This is consistent with presence of large number of dinucleotide repeats in *B. mori* genome (Prasad et al. 2005). Variability in number of markers generated by different primer systems was reported earlier in plant systems as well (Akagi et al. 1996). Association of different ISSR markers with biomass traits was established using MRA. Regression analysis was used to associate molecular markers with economic traits in agricultural crops (Barbosa-Neto et al. 1996; Lynch, 1999; Yonash et al. 2000; He et al. 2002) and for different quantitative traits in *B. mori* (Table 1). In *B. mori*, 45 dominant markers (ISSR—Chatterjee and Mohandas 2003; RAPD—Chatterjee and Pradeep, 2003) and 32 co-dominant markers (RFLP - Sethuraman et al. 2002; STS—Mohandas et al. 2004; AFLP - Gaviria et al. 2006) were found associated with CWT and 26 dominant markers and 15 RFLP markers, with total larval duration. An ISSR marker associated with long larval duration was identified from an inbred population of *B. mori* after artificial selection (Pradeep et al. 2005). Stepwise MRA selected the markers based on its contribution to trait and consequently, interaction and additive effect of multiple markers on a specific trait could be assessed. MRA identified 35 ISSR markers in association with different biomass traits. These markers were correlated negatively or positively with estimates of phenotypic characters. Test of significance on association of markers with different traits reduced number of significant markers to 12. In the first step of MRA, the marker 830.8_1050bp_ was selected for larval weight, cocoon weight, shell weight and floss content. This marker was exclusively present in strains with low estimates of biomass traits and high floss content. Negative association of this marker with LWT and cocoon characters and its positive association with floss content corroborate with negative correlation (R= −0.754) of floss content with LWT/cocoon/shell weight. Such pleiotropic associations of molecular markers with different cocoon characters and yield attributes were illustrated in *B. mori* (for references see Table 1). Identification of several markers for each trait assigns interactive effect of selected independent variables on the dependent variable (Cochran, 1938; Steel and Torrie, 1980), which in turn substantiates multigenic control of the biomass traits (Shibukawa et al. 1986). Single factor ANOVA showed significant association of 830.8_1050bp_ with biomass traits among different strains. In F_2_ generation, the marker 830.8_1050bp_ was segregated at 1:1 ratio. Notedly, in silkworm *B. mori*, recombination occurs only in the homogametic males and is absent in the heterogametic females. Any F_2_ individual can not be homozygous for both maternal and paternal dominant markers on the same autosome (Nagaraju and Goldsmith, 2002). As the ISSR marker on agarose gel is dominant, it could not distinguish a heterozygote. In F_2_, low yielding homozygote individuals with 830.8_1050bp_ marker and high yielding individuals without this marker appeared in equal proportion (1:1). Though major loci for biomass traits are sex-linked is to be analyzed, recent observations indicated distribution of markers thoroughout the Z chromosome and few markers in the W chromosome (Nagaraja et al. 2005). On the other hand, the marker 835.5_1950bp_ for floss content appeared at true Mendelian ratio of 3:1 ratio which indicates dominant nature of this locus and the high floss content in tropical strains. Chi-square values (χ^2^ = 44; *P* < 0.05) revealed significant genetic contribution of the marker 830.8_1050bp_ to LWT, CWT and SR. Association of 830.8_1050bp_ with different biomass traits reflects pleiotropic effect of the locus on various traits with large effect on LWT that showed positive correlation with cocoon weight and shell weight. Correspondingly, significant genetic association of 836.4_2300bp_ with LWT was also noticed. Though SMA showed significant association of 825.9_710bp_ with TLD, 830.8_1050bp_ and 835.5_1950bp_ with floss content and 830.8_1050bp_ with SWT, Chi-square values were insignificant. This indicates that association of these markers is influenced by causes other than genetic factors. A closest marker flanking a QTL may not be tightly linked to a gene (Michelmore, 1995), which may be due to recombination between the marker and QTL (Collard et al. 2005). Further, shell and floss are made of silk proteins, for which amino acid budgeting is made from amino acid pool present in the larval haemolymph. Depending on nutrient quality of mulberry leaf and environmental factors, availability of amino acids in larval haemolymph varies, which significantly affects silk production (Sehnal and Akai, 1990) and thereby influences shell weight and floss content.

Biomass traits showed a switch in phenotypic expression according to presence or absence of markers associated with a specific trait. For instance, shell weight (0.18g) of *GNM* (having the loci 830.8_1050bp_ and 886.5_2000bp_) is intermediate between *Nistari* (having 830.8_1050bp_) (0.13g) and *NB1* (having 886.5_2000bp_) (0.37g). All together, intermediate mean values of different biomass traits showed significant variation from group mean values, revealed interaction of loci on expression of the biomass traits (Table 9). Of the two markers selected for each trait in this study, one of them is negatively correlated and the other is positively correlated. Combined effect of these markers on phenotypes appeared as intermediate as these loci affect characters in opposite directions (Falconer and Mackay, 1996). Such markers are significant as they contributed genetically in opposite directions in two different (temperate and tropic) environments. In F_2_ generation, LWT varied significantly (*P* < 0.0001) between individuals with 830.8_1050bp_ and 836.4_2300bp_ and those without these markers. These loci had opposite effects on LWT in the parents, *Pure Mysore* and *NB1*. Since the QTL and the markers inherited together in F_2_ progeny, mean of the group with the markers significantly varied (*P* < 0.001) from that of the group without the marker. This indicates that the marker loci 830.8_1050bp_ and 836.4_2300bp_ are associated to a QTL controlling LWT, though linkage has to be established. Significant interactions between QTLs were noticed in soybean in which height variation at one locus is conditional upon another specific allele (Lark et al. 1995) whereas ovariole number in *Drosophila* species is under control of sign epitasis of QTLs (Orgogozo et al. 2005). Interaction of QTLs was also reported for different traits in various organisms including number of abdominal bristles and sex comb teeth in *Drosophila* (Long et al. 1995; Tatsuta and Takano-Shimizu, 2006), fruit traits of tomato (Paterson et al. 1991), seed weight in cowpea and mung bean (Fatokun et al. 1992), maize inflorescence traits (Doebley and Stec, 1993), and protein content in soybean (Tajuddin et al. 2003). Notably, intermediate trait values and presence of both negatively and positively correlated markers are characteristics of *B. mori* strains originated in temperate regions of Asia (China and Japan). These strains were either brought to tropical conditions of India or evolved from Japanese/Chinese parents for commercialization. Localized multiplication over a long period might have resulted in allele substitutions, which are common in tropical strains of *B. mori* (Hirobe, 1968; Gamo, 1983). Allele substitutions lead to phenotypic plasticity (Ungerer et al. 2003) as an adaptation in the tropics. In total gene pool of *B. mori* comprising several strains, genetic markers interacted to control expression level of fitness traits according to the needs during adaptation. More over, impact of a locus associated with a specific character could be augmented or weakened by presence of another associated locus. Intermediate phenotype and genetic setup of temperate strains under tropical conditions reflect genetic differentiation of new silkworm populations. By deficiency mapping of QTL affecting longevity in natural population of *Drosophila*, Pasyukova et al. (2000) suggested that QTL contributing to variation in a quantitative trait between two particular strains contribute to variation of the trait in nature, to which present observations on interactions of ISSR loci and its association with biomass traits in *B. mori* strains corroborate.

Larval duration is an exception, which was significantly higher in *Hu204*, in which both 825.9_710bp_ and 835.11_1050bp_ were present. This may be due to small genetic effect of individual QTLs, which are sensitive to the environment (Mackay, 2004). In *B. mori*, larval duration is influenced by loci sensitive to selection (Pradeep et al. 2005) and alleles of juvenile hormone responsive gene (Pradeep et al. 2005), but the intensity of interaction of environment with them is not known. More over, single QTLs could be fractionated into multiple linked QTLs as found in *Drosophila*, effects of which could not be equal on a trait (Pasyukova et al. 2000; Harbison et al. 2004). In insects, larval duration is influenced not only by genetic factors but humoral and environmental cues also (Sehnal, 1985). Impact of marker × environment interaction to determine total larval duration in *B. mori* is to be analyzed in detail.

## Genetic Divergence

Genetic markers represent genetic differences between strains and reveal sites of variation in DNA (Winter and Kahl, 1995; Jones et al. 1997). Several dominant ISSR markers resolved on agarose gel were used for genetic divergence analysis. Earlier studies revealed relative advantages of different algorithms based on grouping of maize inbreds using RFLP data (Ajmone-Marson et al. 1992; Mumm et al. 1994). Silkworm strains used in this study are of Asian origin. It is well known that most of these strains were descent from China in the long past and adapted to diverse climates, point to genetic closeness among them. This indicated a necessity of more than one algorithm to examine genetic divergence within these closely related silkworm populations. Hierarchical cluster analysis grouped low yielding Indian strains and high yielding temperate strains independently. *Nistari* is an original tropical strain of Indian origin and its rearing has been practiced in Ganges river valley since more than a century (Mukherjee, 1912). Though *Pure Mysore* is a tropical, low yielding Indian strain, its origin is not clear. Low genetic distance and clustering of *Pure Mysore* with *Nistari* reflect that these strains are genetically closer. Long association with tropical conditions and stabilization through continuous commercialization made *Pure Mysore* a segregant population of India. Though *Pure Mysore* is adapted for tropical climate, larval duration is longer as in temperate strains of *B. mori.* The marker 825.9_710bp_ selected for TLD was present in all the low yielders but absent in *Pure Mysore*, and other strains of temperate origin. This is consistent with our earlier observation on presence of TLD associated RAPD marker UBC89.5_1500bp_ in *Pure Mysore* and long duration high yielding strains of temperate origin and its absence in tropical low yielding strains (Chatterjee and Pradeep, 2003). Molecular data on the rare RAPD locus and ISSR locus (present observations) associated with larval duration and alleles associated with juvenile hormone responsive genes (Pradeep et al. 2005) supported the presumption that *Pure Mysore* is a hybrid of Chinese and Japanese strains of temperate origin (Datta, 1984). *C’Nichi* is originally a diapausing strain of Japan but adapted to Indian conditions and became a non-diapausing strain. Isolation of *C’nichi* in the dendrograms signified its stabilization as an independent strain after long-term adaptation to tropic climate. High yielding strains, which grouped together, were originated from Japanese or Chinese parental strains and have been used for sericultural activities in India since 1960s. Average genetic distance between Chinese and Indian strains varied significantly (under Sneath and Sokal measure) when compared with that of Chinese-Japanese and Japanese-Indian strains. This indicates segregation and genetic differentiation of those Chinese strains under tropical conditions of India by continuous localized multiplication. This was supported by the observation on ALSCAL matrix that indicated global distribution of genetic characters of Chinese and Japanese silkworm strains and its convergence in India.

Though marker—trait association studies have to be supplemented with linkage analysis, identification of several potential markers that contribute to develop genetic characteristics of silkworm population and reveal genetic divergence within low and high yielding strains, could have potential practical utility in prospective silkworm breeding program.

## Figures and Tables

**Table 1:** Molecular markers associated with different biomass traits of *B. mori*.

Trait	Number of markers positively associated	Number of markers negatively associated	Marker system	References
Total larval duration	06	09	RFLP	Sethuraman et al. 2002
	01	02	ISSR	Chatterjee and Mohandas, 2003
	02	01	RAPD	Chatterjee and Pradeep, 2003
	01	--	RFLP-STS	Mohandas et al. 2004
	03	01	ISSR	Pradeep et al. 2005; Present study
Total Maximum larval weight	13	13	--	--
	10	20	RFLP	Sethuraman et al. 2002
	--	03	ISSR	Chatterjee and Mohandas, 2003
	02	01	RAPD	Chatterjee and Pradeep, 2003
	--	01	RFLP-STS	Mohandas et al. 2004
	01	03	ISSR	Present study
Total Cocoon weight	13	28	--	--
	15	17	RFLP	Sethuraman et al. 2002
	00	04	ISSR	Chatterjee and Mohandas, 2003
	01	01	RAPD	Chatterjee and Pradeep, 2003
	01	--	RFLP-STS	Mohandas et al. 2004
	05*	--	AFLP	Gaviria et al. 2006
	--	01	ISSR	Present study
Total Shell weight	22	23	--	--
	15	18	RFLP	Sethuraman et al. 2002
	00	03	ISSR	Chatterjee and Mohandas, 2003
	01	02	RAPD	Chatterjee and Pradeep, 2003
	01	--	RFLP-STS	Mohandas et al. 2004
	--	01	ISSR	Present study
	05*	--	AFLP	Gaviria et al. 2006
Total Shell ratio	22	24	--	--
	11	12	RFLP	Sethuraman et al. 2002
	01	02	ISSR	Chatterjee and Mohandas, 2003
	01	02	RAPD	Chatterjee and Pradeep, 2003
	01	--	RFLP-STS	Mohandas et al. 2004
	--	02	ISSR	Present study
Total	14	18	--	--

* Type of association (+ or −) not mentioned.

**Table 2:** Origin, diapause behavior and quantitative traits (mean values) of different strains of silkworm *B. mori* used in the study

	1	2	3	4	5	6	7	8	9	10	11	12	13	14
Strains	Nistari	C’nichi	Pure Mysore	GNM	Moria	KA	NB1	NB4D2	NB7	NB18	Hu204	Chinese Golden- 70	Jam-23	Jam -124
Origin	India	Japan	India	China	India	India	Japan	India	India	India	China	China	India	India
Diapause behavior*	ND	ND	ND	ND	ND	D	D	D	D	D	D	D	D	D
TLD (h)	557.	539	626	557	564	602	621	646	599	600	631.	572	594	616
LWT (g)	2.091	2.096	1.817	2.635	2.685	3.518	4.095	3.535	3.4.32	3.611	3.878	3.614	3.835	3.521
CWT (g)	1.020	1.000	1.000	1.190	1.130	1.500	1.710	1.820	1.560	1.570	1.570	1.530	1.700	1.520
SWT (g)	0.130	0.120	0.140	0.180	0.160	0.250	0.370	0.360	0.280	0.270	0.320	0.240	0.280	0.270
SR (%)	13.00	11.99	14.07	15.21	14.69	16.74	21.43	19.56	18.01	17.23	20.41	15.61	16.60	17.560
Floss (%)	6.800	7.090	11.870	8.320	9.170	4.100	5.100	3.464	3.899	4.391	4.121	2.668	3.893	7.438
Silk waste (%)	32.00	43.30	32.50	14.32	31.65	30.38	17.85	11.50	26.00	25.00	22.98	17.88	36.64	22.90

* ND- non-diapausing; D- diapausing.

**Table 3:** Correlation matrix showing interaction among different quantitative traits of *B. mori* strains.

		LWT	TLD	CWT	SWT	SR	FLOSS
TLD	Pearson correlation	0.524					
	Sig. (2-tailed)	0.055					
	Covariance	12.684					
CWT	Pearson correlation	0.945**	0.648*				
	Sig. (2-tailed)	0.000	0.012				
	Covariance	0.203	5.993				
SWT	Pearson correlation	0.915**	0.744**	0.961**			
	Sig. (2-tailed)	0.000	0.002	0.000			
	Covariance	0.057	1.993	0.023			
SR	Pearson correlation	0.855**	0.779**	0.861**	0.962**		
	Sig. (2-tailed)	0.0001	0.001	0.000	0.000		
	Covariance	1.751	68.834	0.674	0.218		
FLOSS	Pearson correlation	−0.783**	−0.215	−0.790**	−0.690**	−0.540*	
	Sig. (2-tailed)	0.001	0.461	0.001	0.006	0.046	
	Covariance	−1.549	−18.293	−0.597	−0.151	−3.896	
S.WASTE	Pearson correlation	−0.480	−0.439	−0.540*	−0.605*	−0.633*	0.313
	Sig. (2-tailed)	0.082	0.116	0.046	0.022	0.015	0.276
	Covariance	−3.219	−126.897	−1.383	−0.449	−15.468	7.383

Correlation is significant at 0.01 level (**) or at 0.05 level (*) (2-tailed)

**Table 4:** Key to primer details, polymorphism, genetic informativeness and markers exclusive to different strains of *B. mori.*

ISSR Primer (UBC)	Sequence*	No. of products	Size (bp) (range)	Number of polymorphic products (% polymorphism)	Mean diversity index (DI)	Mean effective multiplex ratio (EMR)	Maker index (MI)	Products exclusive to:	

								Strains	Size (bp)
807	(AG)_8_T	07	600–1600	06 (85.71)	0.913	5.143	4.695	--	
809	(AG)_8_G	12	950–2200	12 (100)	0.737	12.00	8.848	NB7	2000
810	(GA)_8_T	06	1100–1400	05 (83.33)	0.918	4.167	3.826	--	
811	(GA)_8_C	07	900–2400	07 (100)	0.759	7.000	5.319	--	
812	(GA)_8_A	14	780–2500	14 (100)	0.893	14.00	12.506	C’Nichi	2700
								NB1	2500
								KA	1100
								NB4D2	950
813	(CT)_8_T	08	950–2700	07 (87.50)	0.873	6.125	5.350	C’Nichi	1800
818	(CA)_8_G	06	900–2100	05 (83.33)	0.764	4.167	3.183	--	
825	(AC)_8_T	09	700–3000	07 (77.77)	0.886	5.444	4.825	--	
826	(AC)_8_C	12	820–2600	12 (100)	0.629	12.00	7.559	--	
827	(AC)_8_G	09	1100–2200	07 (77.77)	0.800	5.444	4.355	--	
830	(TG)_8_G	11	780–2000	11 (100)	0.774	11.000	8.523	C’Nichi	1450
834	(AG)_8_YT	13	600–3000	12 (92.31)	0.791	11.077	8.764	C’Nichi	1400
835	(AG)_8_YC	15	600–3500	15 (100)	0.900	15.000	13.500	Hu204	1500
								C’Nichi	1100
836	(AG)_8_YA	15	800–3000	15 (100)	0.895	15.000	13.419	--	
841	(GA)_8_YC	10	500–2600	09 (90)	0.943	8.100	7.638	C’Nichi	1100;1400
844	(CT)_8_RC	05	1100–2250	05 (100)	0.104	5.000	0.520	C’Nichi	1100
851	(GT)_8_YG	07	620–1700	05 (71.43)	0.817	3.571	2.917	--	
857	(AC)_8_YG	09	700–1700	06 (66.67)	0.748	4.000	2.993	--	
862	(AGC)_6_	08	500–1900	08 (100)	0.103	8.000	0.826	--	
864	(ATG)_6_	10	1100–2700	08 (80.00)	0.750	6.400	4.800	--	
873	(GACA)_4_	08	800–1550	07 (87.50)	0.351	6.125	2.153	--	
881	(GGGTG)_3_	12	910–2700	12 (100)	0.842	12.000	10.103	--	
884	HBH(AG)_7_	11	600–2000	11 (100)	0.769	11.000	8.456	--	
885	BHB(GA)_7_	13	700–2100	13 (100)	0.886	13.000	11.512	KA	2000
886	VDV(CT)_7_	15	600–3000	14 (93.33)	0.829	13.067	10.839	Hu204	3000
Total		252	--	233 (92.46)	0.747+	8.713+	6.697+	--	--

* Y = (C,T); R = (A,G); H = (A,C,T); B = (C,G,T); V = (A,C,G); D = (A,G,T);

+ mean values.

DI = 1−∑_pi2_, where *pi* is the allele frequency of the ith allele

EMR = *n_p_*(*n_p_*/*n*), where *n_p_* is the number of polymorphic loci and *n* is the total number of loci.

MI = DI × EMR.

**Table 5:** ISSR markers selected by MRA for different quantitative traits related with biomass in *B. mori.*

Trait	Marker*	Beta**	t-value	Adjusted R^2^	Significance (P)
TLD	825.9	−0.874	5.404	0.738	0.000
	+835.11	0.361	3.032	0.863	0.016
	+825.2	0.270	3.725	0.947	0.007
	+811.3	−0.165	3.041	0.976	0.023
	+807.4	−0.124	4.658	0.995	0.006
LWT	830.8	−0.933	8.957	0.859	0.000
	+851.1	−0.374	3.649	0.930	0.004
	+836.4	0.196	3.834	0.969	0.003
	+886.13	−0.127	3.528	0.986	0.006
	+886.6	−0.097	3.943	0.994	0.004
CWT	830.8	−0.943	8.529	0.878	0.000
	+810.2	0.268	3.640	0.948	0.007
	+844.5	−0.206	4.332	0.984	0.003
	+830.7	−0.191	3.996	0.995	0.007
	+864.7	0.074	6.087	0.999	0.002
SWT	830.8	−0.909	6.559	0.808	0.000
	+834.11	0.359	3.870	0.925	0.005
	+886.5	0.263	4.040	0.974	0.005
	+885.13	−0.146	3.947	0.992	0.008
	+818.1	0.081	3.199	0.997	0.024
SR	830.8	−0.823	4.344	0.641	0.002
	+834.11	0.459	3.255	0.826	0.012
	+884.9	−0.313	3.482	0.927	0.010
	+826.5	−0.200	3.014	0.966	0.024
	+811.4	−0.141	4.842	0.993	0.005
	+827.2	0.063	4.456	0.999	0.011
Floss	830.8	0.812	4.818	0.631	0.000
	+835.5	0.449	2.893	0.771	0.015
	+884.1	−0.471	3.861	0.899	0.003
	+811.3	−0.276	4.515	0.966	0.001
	+830.11	−0.150	4.545	0.989	0.002
	+851.3	−0.074	3.772	0.996	0.007
	+886.4	0.049	4.282	0.999	0.005
Silk waste	881.4	−0.769	4.168	0.557	0.001
	+885.7	0.443	3.108	0.743	0.010
	+825.6	−0.403	5.359	0.927	0.000
	+836.15	−0.245	5.033	0.979	0.001
	+886.6	0.147	4.578	0.993	0.002
	+826.3	0.068	4.185	0.998	0.004

* + indicate stepwise addition of each marker.

** − indicate negative correlation with the estimate of trait.

**Table 6:** Analysis of test of significance of association of markers with biomass related traits in *B. mori* on the basis of markers selected by MRA.

Trait	Marker selected	Strains# with marker		Phenotype estimate (Mean ± SD) of strains with marker		Significance	Type of relation*

		present	absent	present	absent	*P*	
TLD (h)	825.9_710bp_	1,2,4,5, 11	3,6,7,8,9, 10,12,13, 14	594.723 ± 35.549	608.444 ± 21.425	0.07	−
	835.11_1050bp_	6,7,10, 11.	1, 2, 3,4,5,8, 9, 12, 13, 14	613.50 ± 15.022	587.00 ± 34.791	0.071	+
LWT (g)	830.8_1050bp_	1, 2, 3, 4, 5.	6,7,8,9,10, 11,12, 13, 14	2.265 ± 0.378	3.671 ± 0.217	0.00039	−
	851.1_1700bp_	1, 2, 3	4,5,6,7,8, 9,10, 11,12, 13, 14	2.001 ± 0.160	3.487 ± 0.453	3.08 × 10^−6^	−
	836.4_2300bp_	7,11	1, 2, 3,4,5,6,8, 9,10, 12, 13, 14	3.987 ± 0.153	3.033 ± 0.721	0.0024	+
	886.6_1800bp_	1,3,4,9	2,5,6,7,8, 10,11, 12, 13, 14	2.494 ± 0.712	3.439 ± 0.599	0.068	−
CWT (g)	830.8_1050bp_	1, 2, 3, 4, 5.	6,7,8,9,10, 11, 12, 13, 14.	1.068 ± 0.087	1.609 ± 0.108	1.22 × 10^−5^	−
SWT (g)	830.8_1050bp_	1, 2, 3, 4, 5.	6,7,8,9,10, 11,12, 13, 14	0.146 ± 0.024	0.293 ± 0.046	4.74 × 10^−5^	−
SR (%)	830.8_1050bp_	1, 2, 3, 4, 5.	6,7,8,9,10, 11,12, 13, 14	13.792 ± 1.300	18.127 ± 1.93	0.00037	−
	811.4_1800bp_	1, 2, 5, 14	3,4,6,7,8, 9, 10, 11,12, 13	14.310 ± 2.436	17.487 ± 2.368	0.073	−
Silk waste (%)	881.4_2000bp_	2,4,7,8, 9,13,14	1,3,5,6,10,11,12	18.408 ± 5.345	31.800 ± 6.350	0.0011	−
	836.15_800bp_	1,3,4,5, 6,7,8,9, 10,11, 12,14	2,13	23.747 ± 7.181	39.97 ± 4.709	0.059	−
Floss (%)	830.8_1050bp_	1,2,3,4,5	6,7,8,9,10, 11, 12,13, 14	8.65 ± 2.037	4.342 ± 1.334	0.0055	+
	835.5_1950bp_	2,3,5,14	1,4,6,7,8,9, 10,11,12,13	8.892 ± 2.184	4.676 ± 1.680	0.021	+
	811.3_2100bp_	1,2,6,8,9,10, 11,12,13,14	3,4,5,7	4.786 ± 1.676	8.615 ± 2.789	0.064	−
	851.3_1500bp_	1,2,3,4,5,6,7,9, 11,12,13,14	8,10	6.206 ± 2.716	3.927 ± 0.655	0.0351	−

# Serial number of strains (1–14) as mentioned in Table 2;

* −or + correlation with the estimates as derived by MRA.

**Table 7A:** Single factor ANOVA shows association of ISSR marker 830.8_1050bp_ with different traits of *B.mor*i strains.

		Sum of Squares	df	Mean Square	F	Sig.
LWT	Between groups	6.356	1	6.356	80.223	0.000
	Within groups	0.951	12	0.079		
CWT	Between groups	0.940	1	0.940	90.443	0.000
	Within groups	0.125	12	0.010		
SWT	Between groups	0.070	1	0.070	42.893	0.000
	Within groups	0.020	12	0.002		
SR	Between groups	60.425	1	60.425	19.771	0.001
	Within groups	36.675	12	3.056		
FLOSS	Between groups	59.666	1	59.666	23.213	0.000
	Within groups	30.844	12	2.570		
S.WASTE	Between groups	171.060	1	171.060	2.361	0.150
	Within groups	869.498	12	7.458		

**Table 7B:** Single factor ANOVA shows association of ISSR markers with different traits of F_2_ individuals#

Markers	Trait		Sum of Squares	df	Mean square	F	Sig.
830.8_1050bp_	LWT	Between groups	0.722	1	0.722	3.034	0.091
		Within groups	7.847	33	0.238		
	CWT	Between groups	1.064	1	1.064	37.751	0.000
		Within groups	0.930	33	0.028		
	SWT	Between groups	0.021	1	0.021	22.185	0.000
		Within groups	0.031	33	0.001		
	SR	Between groups	0.483	1	0.483	0.070	0.793^ns^
		Within groups	228.222	33	6.916		
	FLOSS	Between groups	223.663	1	223.663	77.652	0.000
		Within groups	95.050	33	2.880		
836.4_2300bp_	LWT	Between groups	0.260	1	0.260	1.304	0.317^ns^
		Within groups	8.308	33	8.308		
835.5_1950bp_	FLOSS	Between groups	29.143	I	29.143	3.321	0.077
		Within groups	289.570	33	8.775		
825.9_710bp_	TLD	Between groups	16589.630	1	16589.630	62.092	0.000
		Within groups	3473.304	13	267.177		

# Traits of F_2_ developed from PM × NB_1_ cross in all cases except for TLD which is from F_2_ of C’nichi × NB_1_.

ns not significant.

**Table 8:** Single marker analysis and Chi-square test of different ISSR markers on F_2_ populations developed from divergent strains of *B. mori*.

Cross	Trait	Marker#	Phenotype estimate (Mean ± SD) of F_2_ individuals where the marker showed:		P value	χ^2^ (Goodness-of-fit)	Significance	Skewed ness towards parent

			Presence	Absence				
PM × NB1 F_2_	LWT	830.8_1050bp_	2.044 ± 0.342	3.129 ± 0.140	1.65 × 10^−13^	44.004	0.095	PM
		836.4_2300bp_	2.905 ± 0.378	2.489 ± 0.513	0.010	46.750	0.057	NB1
	CWT	830.8_1050bp_	0.985 ± 0.250	1.367 ± 0.226	0.0001	47.092	0.042	PM
	SWT	830.8_1050bp_	0.139 ± 0.025	0.220 ± 0.023	7.92 × 10^−11^	36.542	0.104 ns*	--
	SR	830.8_1050bp_	15.224 ± 2.282	16.274 ± 2.693	0.306 ns	46.909	0.055	PM
	FLOSS	830.8_1050bp_	11.020 ± 2.282	06.385 ± 1.351	1.23 × 10^−8^	46.909	0.998 ns *	PM
		835.5_1950bp_	9.482 ± 2.871	6.296 ± 2.527	0.0029	27.484	0.384 ns*	--

C’NICHI × NB1 F_2_	TLD	825.9_710bp_	533.714 ± 11.586	600.375 ± 19.522	3.899 × 10^−6^	20.728	0.109 ns*	--

# Inheritance of all markers was at 1:1 ratio except 835.5_1950bp_ was at 3:1; ns: not significant

**Table 9:** Effects of interaction of markers on estimates of biomass traits in *B. mori.*

Traits	Marker combination	Strains in which the marker is present	Mean (± SD) Each strain	phenotype estimate of: Group	Significance
CWT	830.8_1050bp_	Nistari	1.02		
		PM	1.0		
		Moria	1.13	1.05 ± 0.070	0.074*
	830.8_1050bp_ + 810.2_1350bp_	GNM	1.19	1.19#	0.059**
	810.2_1350bp_	NB1	1.71		
		Chinese golden 70	1.53	1.62 ± 0.127	0.131***^ns^
SWT	830.8_1050bp_	Nistari	0.13		
		C’nichi	0.12		
		PM	0.14	0.130 ± 0.01	0.094*
	830.8_1050bp_ + 886.5_2000bp_	GNM	0.18		
		Moria	0.16	0.17 ± 0.014	1.6 × 10^−5^**
	886.5_2000bp_	KA	0.25		
		NB1	0.37		
		NB4D2	0.36		
		NB7	0.28		
		NB18	0.27		
		Chinese Golden-70	0.24		
		Jam23	0.28		
		Jam124	0.27	0.290 ± 0.048	0.0053***
Silk waste (%)	881.4_2000bp_	GNM	14.32	14.32#	0.042*
	881.4_2000bp_ + 885.7_1200bp_	NB1	17.85		
		NB7	26.00		
		Chinese golden 70	17.88		
		Jam124	22.90	21.575 ± 4.007	0.0055**
	885.7_1200bp_	Jam 23	36.64		
		C’nichi	43.30		
		Moria	31.65		
		KA	30.38	35.493 ± 5.864	0.009***
Floss (%)	830.8_1050bp_	Nistari	6.80		
		PM	11.87		
		GNM	8.32		
		Moria	9.17	9.04 ± 2.126	0.164* ns
	830.8_1050bp_ + 830.11_780bp_	C’Nichi	7.09	7.09#	0.0078**
	830.11_780bp_	NB4D2	3.464		
		Chinese golden 70	2.668	3.066 ± 0.563	0.063***
TLD	825.9_710bp_	Nistari	557		
		C’nichi	539		
		GNM	557		
		Moria	564	554.25 ± 10.689	0.0007*
	825.9_710bp_ + 835.11_1050bp_	Hu204	631	631#	0.0028**
	835.11_1050bp_	KA	602		
		NB_1_	621		
		NB18	600	607.667 ± 11.590	0.072***
LWT	830.8_1050bp_	Nistari	2.091		
		C’Nichi	2.096		
		PM	1.817		
		GNM	2.635		
		Moria	2.685	2.265 ± 0.378	
	830.8_1050bp_ + 836.4_2300bp_	Nil$	--	--	
	836.4_2300bp_	NB_1_	4.095		
		Hu204	3.878	3.987 ± 0.153	0.0046**

* Significance of difference between low and intermediate estimates.

** Significance of difference between low and high estimates.

*** Significance of difference between intermediate and high estimates.

$ No strain had both markers together; ns- not significant;

# single strain.

**Table 10:** Genetic distance between different strains of *B. mori* of different geographical origin based on ISSR profile derived using three different measures.

	Jaccard measure		Dice measure		Sokal and Sneath measure	

	Strain	Genetic distance*	Strain	Genetic distance*	Strain	Genetic distance*
Most genetically similar pair	Nistari- Pure Mysore	0.108	Nistari- Pure Mysore	0.216	Nistari- Pure Mysore	0.108
Most genetically distanced pair	C’Nichi- Jam23	0.667	C’Nichi- Jam23	0.500	C’Nichi- NB1	0.292
Mean (range in parenthesis) genetic distance between Chinese and Indian strains	--	0.539 (0.411–0.632)	--	0.371 (0.259–0.462)	--	0.193** (0.118–0.238)
Mean (range in parenthesis) genetic distance between Chinese and Japanese strains	--	0.556 (0.513–0.617)	--	0.386 (0.345–0.446)	--	0.220 (0.180–0.263)
Mean (range in parenthesis) genetic distance between Japanese and Indian strains	--	0.559 (0.440–0.667)	--	0.390 (0.282–0.500)	--	0.225 (0.108–0.292)

* calculated from similarity matrix;

** Significant at p < 0.01 level when compared with genetic distances within Chinese-Japanese and Japanese-Indian Strains.
